# Exercise’s impact on lung cancer molecular mechanisms: a current overview

**DOI:** 10.3389/fonc.2024.1479454

**Published:** 2024-11-01

**Authors:** Annamaria Mancini, Francesca Maria Orlandella, Daniela Vitucci, Neila Luciano, Andreina Alfieri, Stefania Orrù, Giuliana Salvatore, Pasqualina Buono

**Affiliations:** ^1^ Department of Medical, Human Movement and Well-being Sciences, University Parthenope, Naples, Italy; ^2^ CEINGE-Biotecnologie Avanzate “Franco Salvatore”, Naples, Italy; ^3^ Department of Advanced Biomedical Sciences, University Federico II, Naples, Italy

**Keywords:** exercise, lung cancer, tumor microenvironment, angiogenesis, gene expression, apoptosis, intermediate metabolism

## Abstract

Lung cancer is the major cause of cancer-related deaths worldwide with an estimated 1.8 million deaths and 2.4 million new cases in 2022. Poor cardiorespiratory fitness, dyspnea and fatigue are the common features in lung cancer patients, partially limiting the exercise prescription. Exercise improves cardiorespiratory and muscular fitness and reduces the risk of some types of cancer, including lung cancer. Recently, the American Society of Clinical Oncology has encouraged preoperative exercise for lung cancer patients. Nonetheless, only limited data, mostly obtained from mouse models of lung cancer, are available on the molecular effects of exercise in lung cancer. Thus, the present minireview aims to shed light on the molecular mechanisms induced by different type of exercise in lung cancer. In particular, the role of the exercise in tumor microenvironment remodeling, angiogenesis, gene expression, apoptosis and intermediate metabolism will be examined.

## 1 Introduction

Globally, in 2022 the incidence of Lung Cancer (LC) is estimated of 2.4 million new cases representing, the first and the second most commonly diagnosed malignancy in men and women, respectively. Moreover, with 1.8 million estimated deaths in 2022, this tumor is the first cause of cancer death in men and the second cause in women (1, 2).

The most frequent form of LC is represented by the non-small cell lung cancer (NSCLC). NSCLC further encompass two major subtypes: lung adenocarcinoma (LUAD) and lung squamous cell carcinoma (LUSC). Small cell lung cancer (SCLC) includes approximately 15% of LC cases and is characterized by high proliferative rate, mutational burden and poor survival. The pathogenesis of LC, in particular the SCLC subtype, is mainly due to environmental factors as smoke (3, 4). The tumor is highly heterogenous from a genetic, metabolic and immunological point of view and both spatially and temporally. This heterogenicity has important implication in therapy resistance (5–8).

The therapeutic options for LC patients depend on the tumor stage, grade, histological subtype and the overall condition of the patient. Among the options surgery, chemotherapy and radiotherapy are included; however, LC patients treated with these standard procedures often relapse. Generally, almost all patients with SCLC will relapse, while for NSCLC the percentage of relapse is approximately of 30-50% (9). In the recent years progress in basic cancer research, allowing the identification of key genetic lesions, has improved the scenario therapy for LC patients (10–12). A range of inhibitors targeting key genes are currently available for LC treatment (13).

Immunotherapy is also being used for these patients. The use of antibodies targeting the programmed death receptor (PD-1), its ligand (PD-L1), and the cytotoxic T-lymphocyte-associated protein 4 (CTLA-4) receptor, has improved the survival of patients with NSCLC (14, 15).

Recently, the practice of exercise in cancer patients has gained increasingly attention with potential advantages beyond traditional treatments (16–18). In LC, the use of exercise is in part limited, mainly due to dyspnea being a major symptom. However, the role of exercise preoperatively has been described (19, 20). The American Society of Clinical Oncology has encouraged preoperative exercise in LC patients, for better recovery and minimizing complications after surgery (21–23). In addition, an active lifestyle should be recommended during cancer treatment to help patients not only maintain muscle mass and function but also to promote long-term health (24). As for the postoperative period, structured rehabilitation programs, supervised by professionals, play a key role in ensuring safe exercise after lung surgery, leading to a better recovery. Exercise not only could prevent complications like pneumonia but also could improve emotional health, reducing anxiety and depression. Additionally, it enhances muscle strength and oxygen efficiency, which reduces cardiovascular and pulmonary strain (25). Nevertheless, besides these strong evidences, precise prescription of exercise for people with LC is currently unknown and guidelines are lacking.

**Figure 1:**
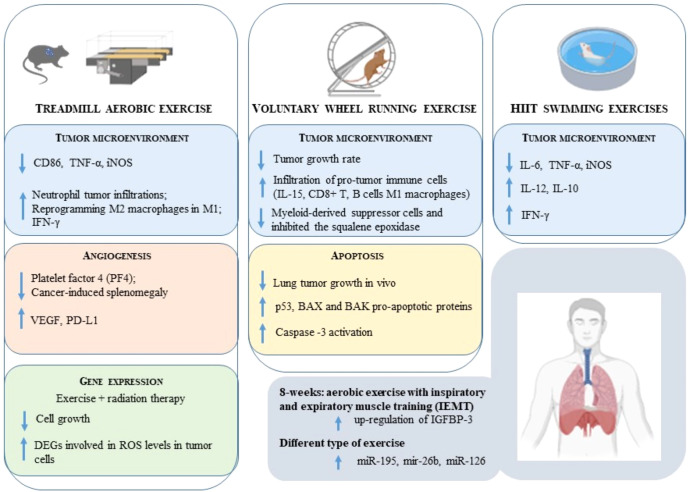
A graphical summary of the molecular mechanisms elicited by the exercise in LC.

This minireview has the focus of giving an update insight on the molecular mechanisms induced by exercise in LC, contributing to current knowledge on the topic (26, 27). In particular, the role of exercise in tumor microenvironment, angiogenesis, gene expression, apoptosis and intermediate metabolism will be dissected. A summary of the main findings emerged from the literature is presented in **Figure 1** and **Table 1**.

**Table 1:** Exercise-induced molecular changes in LC.

Author and year	Study Design	Target group	Exercise intervention description	Main results
Mouse models				
Alves et al., 2018 (28)	Experimental groups: sedentary mice (LLC group) or HIIT (LLC + HIIT group) n=6-8	C57BL/6 mice injected with LLC cells	Adaptation: 10 min for 5d of running on treadmill (15° inclination at <15 m.min^-1^ speed). Then, mice were injected with LLC cells and HIIT training protocol (16d) started: 5x3min at 18 m.min^-1^ speed + 4min at 25 m.min^-1^ speed. At 18°d mice underwent maximal incremental running test: running until exhaustion at 15° inclination at 6 m.min^-1^ speed incremented of 3 m.min^-1^ every 3min.	LLC + HIIT group had lower tumor mass than LLCgroup; HIIT increased Cd274 (PD‐L1) mRNA expression and Vegfa mRNA expression
Ge et al., 2022 (29)	Experimental groups: control group (n=15), Exercise group (n=15), HIIT group (n=15)	Mouse model of LC (6-w-old specific-pathogen-free ) grade BALB/c female mice)	Exercise and HIIT group mice performed 12 w of exercise. Exercise: endurance exercise with an intensity of 15m/min (80% VO_2_max) for 45min, 5 times/w. HIIT: swimming exercises with a lead mass at mice tail for 10% (for the first 6w) or 12% (in the last 6w) of body weight. Mice swam for 20sec + 10sec recovery x 10 times x 4 times/w.	Endurance exercise reduced the proportion of M1-type TAMs in lung cancer tissues HIIT antagonistically regulated M1 and M2 polarization of TAMs by increasing the levels of IL-10 and IL-12 in LC tissues and circulating IFN-γ
Higgins et al., 2014 (30)	Experimental groups: no activity (n=10), exercise (n=10)	Lung Adenocarcinoma Mouse Xenografts	28d -period of voluntary wheel running exercise. Exercise was monitored by a digital wheel revolution counter and km ran were assessed. Sedentary mice cages were not provided of running wheels.	Exercise: repressed lung tumor growth *in vivo*, induces the up regulation of p53 and apoptosis in tumor tissues
Jo et al., 2024 (31)	Animals were subsequently subjected to aerobic exercise and radiation 3 times/w for 2w (n=5 per group).	NSCLC xenograft-mouse model	Combined aerobic exercise and radiation therapy group performed aerobic exercise on a treadmill at 8.0 m/min speed for 30 min 3 times/w for 2w, followed by radiation.	Aerobic exercise improved the effectiveness of radiation in the treatment of NSCLC
Leimbacher et al., 2023 (32)	Mice injected with LLC1.1 cells carry out a voluntary wheel running exercise (running, n =8; not-running, n =8)	C57Bl/6 mice injected with LLC1.1 cells	Running mice group performed moderate intensity voluntary running exercise on a wheel in cages with an open running wheel before (d 0–30) and after LLC1 implantation (d 31–48).	Voluntary exercise does not suppress invasion and growth of LLC1.1 lung cancer cells and does not increase muscle-derived IL-6 levels
Luo et al., 2023 (33)	LLC mouse model or control carry out a voluntary wheel running exercise (sedentary, n =5; trained, n =5)	C57BL/6 mice injected with Lewis mouse NSCLC cell	Voluntary wheel running exercise. Additional information was NR.	Exercise inhibited the infiltration of pro-tumor immune cells, such as myeloid-derived suppressor cells and inhibited the squalene epoxidase, correlated with immuno-cold tumour microenvironment
Martín-Ruiz et al., 2020 (34)	Experimental groups: non-exercise + isotype control (n=5), exercise + isotype control (n=5), non-exercise + nivolumab (n=6) exercise + nivolumab (n=6)	Mouse model for (PDX) of NSCLC	8-w moderate-intensity training regimen (treadmill aerobic exercise and strength training). Aerobic training 5 d/w for 30-40min: mice started with very low workloads (20min at 40% of the maximal velocity obtained previously) and ended with 40min at 80% of maximal velocity and 15% gradient in the last sessions+ maximum forelimb grip strength measuring maximal isometric force. The strength training was performed after the aerobic training, 2/w and included: horizontal screen exercise and hanging exercise with two limbs.	Exercise alone reduced the tumor growth rate Double intervention (exercise + nivolumab) increased tumor necrosis and reduced apoptosis
Tobias et al., 2023 (35)	C57BL6 and Balb/c mice were injected with tumor cells and subjected to different exercise protocols (sedentary, n = 12; trained, n = 11)	C57BL6 mice injected with B16F10 or LLC tumor cells Balb/c mice injected with CT26 or 4T1 tumor cells	C57BL6 mice: voluntary running wheels for 60d (45d before tumor cells injection + 15d after tumor cells injection). Balb/c, Balb/c mice: treadmill training protocol 6w of running (4w before tumor cell injection + 15d after tumor cell injection), 5 d/w, for 60min at 60% of the maximal intensity)	Exercise reduced cancer-induced splenomegaly decreasing platelet factor 4 mRNA levels in the CT26 tumor cells.
Human				
Kurgan et al., 2017 (36)	Exposure of NSCLC cells to post exercise serum	Recreationally active male university students subjected to HIE	HIE cycle ergometer protocol done on a 2-visits period. 1° visit: 5min cycling at 80W followed by incremental test with resistance increased by 15W every min until exhaustion, maximal workload was recorded. 2° visit: HIE trial based on cycle ergometer at 90% workload consisting in 6 x 1min HI cycling intervals separated by 6 x 1min active rest periods. Protocol also constituted by 4min warm-up and 2-3min cool-down cycling at 70W.	inhibition of cell proliferation and survival, reduction of phosphorylated/activated Akt, mTOR, p70 S6K, and Erk1/2 levels compared to cells treated with pre-exercise serum
Liu et al., 2022 (37)	miRNAs expression datasets related to LC and exercise were collected to screen altered miRNAs.	The miRNA and mRNA expression profiles of LC and exercise from Homo sapiens datasets	The authors summarize all the data collected into a single definition of exercise: regular exercise. Additional information was NR.	The results identified 42 marker miRNAs in LC, of which three core-miRNAs (has-miR-195, has-miR-26b, and has-miR-126) were coregulated by exercise and cancer, mainly involved in cell cycle and immunity.
Messaggi-Sartor et al., 2019 (38)	A two-center prospective, single blind, pilot randomized control trial (exercise program, n=16; usual care, n=21)	Patients newly diagnosed with resectable NSCLC	Combination of 2 exercise modalities: continuous aerobic training + inspiratory and expiratory muscle training (1h-session x 3 times/w x 8w): 30 min aerobic training done on ergometric bicycle at 60% workload, increased by 5W weekly + 5 sets x 10 rep followed by 1-2min recovery, done twice/d 3 d/w x 8w.	up-regulation of IGFBP-3

d, day; HIE, high intensity exercise; HIIT, high intensity interval training; LC, Lung cancer; LLC, Lewis LC Lung cancer; m.min, meters.minutes; NSCLC, non-small cell lung cancer; NR, not reported; PDX, Patient- derived xenograft; rep, repetitions; TAM, tumor-associated macrophage; W, Watt; w, week.

## 2 Effects of exercise on tumor microenvironment

The tumor microenvironment is a complex and dynamic entity composed by a great variety of elements as immune and stromal cells, macrophages, extracellular matrix and blood vessels (39). To date, the relationship between exercise and tumor microenvironment has been elucidated in different types of cancer as breast (40), pancreatic (41), melanoma (42) and hepatocellular (43) carcinoma. Mechanistically, exercise is reported to be able to remodel the tumor microenvironment through the modulation of immune system (44). For example, in pancreatic cancer, following aerobic exercise an anti-tumor immune cells redistribution occurs characterized by an accumulation of interleukin (IL)-15 and CD8+ T cells in the tumor microenvironment, which are hurdle against tumor growth (41). Also in breast cancer, the potential anticancer effect of exercise is due to an accumulation of CD8+ T cells into tumor microenvironment (40).

However, despite the growing literature on the role of exercise in this context, if and how exercise could affect the tumor microenvironment in LC was only partially investigated. To our knowledge, the first evidence that the exercise intervention could modulate the tumor microenvironment in LC was highlighted by Martín-Ruiz and colleagues in 2020 investigating the effects of combination of exercise and immunotherapy in mice subcutaneously injected with cancer cells derived from NSCLC patient (34). After 8-week of moderate intensity exercise training, the authors evidenced that exercise alone reduced tumor volume and increased neutrophil tumor infiltrates. The combined treatment (exercise and therapy) increased tumor necrosis (34).

The benefits of exercise on tumor microenvironment were also reported in a recent preclinical study performed in a C57BL/6 mice injected with Lewis LC (LLC) mouse model (33). Mice after 20 days of voluntary wheel running, presented a reduction in tumor volume and a higher infiltration of CD8+ T cells, B cells and M1 macrophages compared to the group of non-exercise mice. Further in this LC mouse model, lower concentration of myeloid-derived suppressor cells was observed that, contrariwise, exert a protumor activity. Interestingly, the aerobic exercise training inhibited the squalene epoxidase, an enzyme involved in the reprogramming of cholesterol metabolism notably correlated with immuno-cold tumor microenvironment (33).

Also the link between the exercise and the polarization of tumor-associated macrophage (TAM) was analyzed in a mouse model of LC (29). The endurance exercise induced a reprogramming of M2 macrophages in M1 type, as witnessed by a reduction in the expression of CD86, Tumor necrosis factor (TNF)- α and nitric oxide synthase (iNOS) markers, delaying tumor growth. The authors also investigated the role of high-intensity interval training (HIIT), an anerobic exercise characterized by high-intensity peak (30 sec) interspersed by low-intensity (recovery) efforts (10 sec) (45). HIIT exerts an anti-inflammatory effect since occurred a reduction in cancer tissues of the expression of IL-6, TNF- α and iNOS and an increase in the level of IL-12 and of IL-10 in the HIIT mice group. Interestingly, in the blood of mice performing endurance or HIIT exercise, the circulating levels of interferon gamma (IFN-γ) were increased compared to the non-exercise mice group (29). Overall, these data suggested that benefits of exercise in LC could be also ascribed to the reprogramming of TAM and to the modulation of inflammatory markers cytokines expression.

In contrast, Leimbacher and colleagues asserted that tumor nodules from running mice had greater immune cell infiltration than those from non-running mice. Therefore, exercise improves the oxygenation in the lungs and tumor nodules by reducing anemia, but it did not influence either the lung invasion or the proliferation of LLC1 tumor cells (32).

## 3 Effects of exercise on angiogenesis

The intra-tumoral hypoxia, through the activation of several genes expression, enhanced the aggressive behavior of cancer cells and hindered the delivery of drug contributing also to therapy resistance (46). Thus, the knowledge of the effects of exercise intervention on angiogenesis is an important step in the oncology field that could improve the therapeutic response.

Emerging data showed that the improvement of blood vessels could be obtained by acute and chronic aerobic exercise training contributing to a better perfusion and vascularization of tissue (47). However, the molecular pathways underlying the link between exercise and angiogenesis remain poorly understood. For example, it was reported that exercise training increased the recruitment of endothelial progenitor cells from the bone marrow in the blood inducing vascular normalization (48). Moreover, it has been evidenced that exercise could affects breast, prostate and hepatocellular tumor growth and the aggressiveness of these cancer cells by improving tumor angiogenesis through reduction of intra- tumoral hypoxia and enhancement of blood perfusion (49–51). Mechanistically, aerobic exercise is able to increase the expression of several angiogenetic factors, like Hypoxia inducible factor (HIF)-1, ANGIOPOIETINS, Vascular endothelial growth factor (VEGF), Platelet derived growth factor (PDGF), Fibroblast growth factor (FGF) that exert a pro-angiogenetic effect (52).

The relationship between exercise and angiogenesis is also supported in mice bearing hepatocellular carcinoma where the swimming intervention impaired hypoxia through the inhibition of HIF-1α and serine/threonine kinase 1 (AKT)/Glycogen synthase kinase 3 beta (GSK-3β)/β-CATENIN signaling pathways (51).

Besides these evidences in different types of cancer, molecular studies concerning the link between exercise and tumor angiogenesis in LC are lacking.

Recently, the potential therapeutic effects of exercise intervention in LC were suggested by Tobias and colleagues evidencing that aerobic exercise reduced the tumor growth and cancer-induced splenomegaly in Lewis LC bearing mice by decreasing the expression of platelet factor 4 (PF4), a protein correlated to vessel formation (35).

Also, higher levels of VEGF and of PD-L1 were found in mice bearing Lewis LC after HIIT, compared to sedentary tumor mice, that counteract tumor progression by angiogenesis promotion (28).

## 4 Effects of exercise on gene expression

A growing body of evidence shows that regular endurance and resistance training can induce changes in gene expression profiles by epigenetic mechanisms (53–55). In addition, it has been found that exercise modulates the expression of microRNAs (miRNAs), their release into circulation and their target genes (17, 56–58).

Recent studies revealed alterations in miRNAs expression in LC patients in response to exercise. For instance, Liu and colleagues observed that exercise increased the expression of miR-195, miR-26b and miR-126, which are normally down-regulated in LC. These miRNAs affect biological processes like immune response modulation, tumor suppression and cell cycle regulation. Moreover, differential expression of circulating miR-195, miR-26b and miR-126 and their target genes have been linked to a worse prognosis in LC patients (37).

Recent research focused on unraveling the genes whose expression resulted modified by aerobic exercise in combination with radiotherapy in a xenograft mouse model of NSCLC. The authors found that the combination of exercise with radiation therapy reduced cell growth. Transcriptomic analysis was performed on tissues derived from the treated mice. Differentially expressed genes (DEGs) were analyzed using the Kyoto Encyclopedia of Genes and Genomes (KEGG) and gene ontology (GO), revealing a strong correlation among the different expression of most of DEGs and angiogenesis, vascular and miRNAs. Notably, four genes associated to the ROS pathways, i.e., Glutathione S-transferase mu 5 (GSTM5), mitochondrial permeability transition pore (MPTP), Glutathione S-transferase omega 1 (GSTO1) and solute carrier family 25 member 31 (SLC25A31), showed different expression between the radiation-alone and combination groups. This preclinical study suggests that combining aerobic exercise with radiation therapy may improve LC patients’ treatment outcomes (31).

In summary, these data highlight the potential of aerobic exercise associated to radiation therapy as an effective modulation of cell growth and gene expression in LC patients. However, the exact mechanisms by which exercise affects gene expression in LC patients should still being elucidated.

## 5 Effects of exercise on apoptosis

Autophagy and apoptosis are crucial processes in LC progression, promoting self-regulatory mechanisms in response to cellular stress and death signals (59, 60). The effects of the exercise on cancer cell apoptosis have been confirmed by numerous studies, supporting the beneficial effects as adjuvant therapy, promoting suppression of tumor growth and cancer cell apoptosis (24, 61, 62). Kurgan and colleagues show that serum from a post- high intensity exercise (12 intervals at 90% workload) is able to inhibit proliferation and survival of LC cells, in agreement with previous studies that demonstrated similar effects from a single session of moderate-intensity aerobic exercise (20 minutes at 50% or 40 minutes at 65% of VO2 max) on prostate cancer cells (63), as well as a single session of combined HIIT and resistance training (30 minutes warm-up, 60 minutes of resistance training, and 30 minutes of high-intensity interval spinning on stationary bicycles non è specificato intensità) on breast cancer cells (64). The authors also evidenced that the mechanism underlying this inhibition was due to the phosphorylation/activation of AKT, mechanistic target of rapamycin kinase (mTOR), P70 (ribosomal protein S6 kinase B1) S6K and mitogen-activated protein kinase (ERK)1/2 signaling pathways (36).

The tumor suppressor p53, that was found mutated in 50% of NSCLC, maintains genomic integrity by responding to cellular stress and DNA damage through promotion of cell cycle arrest and DNA repair or apoptosis (30, 65, 66). Higgins and colleagues demonstrated that p53 protein levels were strongly increased in LC of aerobic exercising mice compared to that of sedentary mice. In addition, levels of the pro-apoptotic proteins BCL2 associated X, apoptosis regulator (BAX) and BCL2 antagonist/killer 1 (BAK) were significantly higher in LC tissue from exercising than in sedentary mice tumor, indicating that p53-driven apoptosis occurs in exercise. Similarly, increased levels of the apoptotic intermediate, active CASPASE-3, were found in LC of aerobic exercising compared with sedentary mice tumor. These results suggest that aerobic exercise reduces the cancer growth through the increase of p53 expression and subsequent p53-driven apoptosis (30).

The BECLIN-1 protein is crucial in autophagy initiation and tumor suppression (67). The loss of BECLIN-1 slows-down the autophagy pathway and in turn potentially increases the carcinogenesis by preventing the degradation of harmful agents (59, 67). BECLIN-1, through the interaction with BCL-2 protein, also promotes the release of pro-apoptotic molecules, like BAX and BAK in some type of cancer cells. Additionally, the (ATG)5-ATG12-ATG16 complex, involved in the autophagy pathway activates the apoptosis through ATG12’s interaction with anti-apoptotic BCL-2 proteins, underscoring the complex interplay of autophagy and apoptosis in cancer development (60, 68, 69).

Finally, imbalance of autophagy also plays an important role in cancer cachexia (70). Cachexia occurs in about 50% of patients with lung cancer (71).

Recent research focused on the effects of the endurance and resistance exercise in the induction of proteins, involved in the autophagy pathway, like ATG5, p62, pULK1, in association to cancer cachexia (70, 72–74).

In conclusion, the interaction between exercise-induced apoptosis and LC results intricates yet crucial. Aerobic exercise induces the activation of p53-tumor suppression protein expression in LC mouse models; similarly, exercise increases the expression of BAX and BAK and CASPASE-3 proteins, and as suggested in other types of cancer, exercise could influence the expression of BECLIN-1, a bridge protein, linked to autophagy pathways and other proteins associated to the cachexia.

## 6 Effects of exercise on intermediate metabolism

Energy balance is essential for maintaining cell survival and overall body stability. The AMP-activated protein kinase (AMPK) acts as cellular energy sensor, regulating different signals and metabolic pathways in response to different stimuli, such as obesity (75), aging (29), caloric restriction (76) and exercise (77). AMPK activation can influence different cellular processes, including cell proliferation, apoptosis and the response to oxidative stress (78). Dysregulation of AMPK signaling is a common finding in several cancer types, including LC (79). In recent years, AMPK activation has emerged as a promising therapeutic target for different types of cancer due to its role in cell proliferation and energetics. It is widely recognized that AMPK activation occurs in response to muscle contraction and exercise (80–82). Exercise, as well as other stressful factors such as fasting (83), determines changes in the expression levels of several hormones that activate AMPK and their intracellular signaling pathways to maintain cellular and systemic energetic homeostasis (84). Acute exercise induces AMPK activation (85), which in turn inhibits cancer cell growth and promote protective autophagy in cells activating liver kinase B1 (LKB1), the upstream activator of AMPK (86). Furthermore, AMPK activation improve tissue insulin sensitivity (85). Insulin and insulin-like growth factor (IGF)-1 play important role also in glucose metabolism and cell proliferation. Increased expression of IGF-1 has been described in association with increased risk of different type of cancer (87) although the molecular mechanisms are not completely elucidated. Lung fibroblasts have been shown to synthesize IGF-1 (88). IGF-1 plays a critical role in lung disease, like cancer and lung fibrosis (89); interestingly, the expression of IGF-1 in LC tissue was higher than in adjacent normal lung tissue (90). The IGF-1 receptor (IGF-1R) is a central component of LC signal transduction pathways (91). Overexpression of IGF-1R was reported in NSCLC and SCLC, by Long et al., that evidenced after intrasplenic injection in mice of LC cells and IGF-1R receptor, an increase in the metastatic activity (92). On the contrary, downregulation of IGF-1 and upregulation of IGFBP-1, achieved through diet and exercise, may have protective effects against cancer cell development, depending on the type, intensity, and duration of training (93). The first exercise intervention study involving surgically treated LC patients who had not undergone adjuvant chemotherapy or radiotherapy, resulted in up-regulation of IGFBP-3. The exercise intervention included an 8-week training aerobic exercise combined with inspiratory and expiratory muscle training improved the response to the treatment in the human (38). In conclusion, the exercise contributes to the reprogramming of intermediate metabolism in cancer cells/tissue through the activation of AMPK, IGF-1 and IGF-1R resulting in a potential nonpharmacological adjuvant in the management and treatment of some types of cancer including LC. However, the molecular mechanisms are not completely elucidated until now.

## 7 Conclusive remarks

This minireview addressed some topics on molecular effects mediated by exercise in LC highlighting the evidence obtained in this field, mostly in mouse LC models, pointing-out the existing gap in knowledge on the molecular effects of exercise in human LC, which still requires further investigations.

Overall, these evidences support the positive impact of the exercise in LC and underline the poorly understood molecular mechanisms elicited in human.

The most relevant results discussed are summarized in **Figure 1**.

## 8 Limitations and future perspectives

The focus of this minireview is to provide an updated overview of the impact of exercise on molecular mechanisms in LC. Due to the limited molecular data on the effects of exercise in LC patients, we primarily reported and discussed on animal models. This reliance on animal models limits the direct applicability of the findings to humans. Human clinical trials are essential to confirm the therapeutic potential of exercise and to validate the translatability of preclinical results. Furthermore, the heterogeneity in exercise protocols, including different types, frequency, intensity, time, volume, and progression, complicates the development of specific guidelines for clinical application. Personalized exercise prescription, adapted to the different clinical stages and specific to the type of oncological pathology, is needed to optimize and replicate therapeutic outcomes across different patient populations. Future human studies should focus on detailed mechanistic investigations, which could help in the development of targeted exercise interventions.
